# Medical and Physical Intelligence

**Published:** 1825-03

**Authors:** 


### MEDICAL AND PHYSICAL INTELLIGENCE.

### ANATOMY.

1. *On the Cochlea of the Internal Ear of Birds.*—THE following letter is from the pen of Dr. TREVIRANUS, of Bremen:—

The structure of the cochlea of the internal ear of birds, has been hitherto principally known by the description of Professor SCARPA; from which it appears so simple, that it is rather difficult to comprehend how a class of animals, in which many species display an acuter sense of melody and of articular tones than most of the mammalia, should, in respect to the perfection of this organ, which certainly is principally intended for the reception of the different modifications of audible impressions, be so far inferior to them. According to Scarpa, this part contains only a hollow cartilage, in which the nerves of the cochlea terminate. I have always considered this description as imperfect, and have found my opinion confirmed by careful examination of the internal ear of different birds. In the cochlea of the Falco *lagopus*, Corvus *glandarius*, Ardea *stellaris*, Fringilla *canaria*, and Loxia *coccothraustes*, I discovered, beneath a cuticular disc or cover, a double row of cuticular folia, on the walls of which the greater portion of the cochlear nerve is distributed, while only one branch of it is distributed to the hollow cartilage of Scarpa. On the contrary, the cochlea of the domestic fowl had none of these folia, but was simple in its structure. In the duck, the foliated arrangement was present, but not so decidedly marked as in the birds already enumerated. Probably the same is the case in the goose, but of this I have not satisfied myself by actual examination. But Scarpa appears to have examined only the cochlea of this latter bird, and hence we can explain how the structure we have pointed out has escaped his attention.

Those birds which are remarkable on account of their acuteness of hearing, are those also in which the cochlea is provided with perfectly-formed cochlear plates. By means of the great number of these plates, a wider space is afforded for the distribution of the cochlear nerve, and probably the space is proportionally greater in birds than in the lamina spiralis of the cochlea in quadrupeds.

It is my intention soon to publish a full account of the fact, because a detailed description, without drawings, would not be sufficiently intelligible. I may add, for the information of those who may wish, by actual investigation, to satisfy themselves of the accuracy of my statement, that, in order to see the cochlear plates or folia, and the distribution of the cochlear nerve, it is necessary to harden the ear in spirits of wine, before dissection.—*(Edinburgh Phil. Journal.)*

2. ***Discovery of a Lymphatic Vessel opening into the Vena Cava inferior.***—Dr. Regolo Lippi, lecturer on human and comparative anatomy, at the hospital of St. Mary, in Florence, discovered, on the 24th of April, 1824, a large lymphatic trunk, which emptied itself into the vena cava inferior, opposite to the third lumbar vertebra. This vessel traversed the coats of the vein, which it penetrated in a direction opposite to the course of the blood. It was furnished, at the point of union with the vein, with a valve, which was very perceptibly distended with mercury when this vessel was filled, and an examination of its internal communication with the vein was made.

Another set of injections was made on the 27th of April, when they succeeded in filling four lymphatic trunks, all communicating with the inferior cava. One of them opened into the common iliac, while the three others emptied themselves, as in the foregoing case, into the inferior cava.

Having injected a liver that had been affected with inflammation, and the injection having penetrated to the surface of this viscus, Dr. Lippi perceived several lymphatic vessels of the triangular ligament enter the ramifications of the vena portæ.

Since, however, this first injection had been used on the right side, he wished to make a second on the left: he therefore threw the mercury into the external iliac lymphatic. The injection now penetrated to those vessels which are situated behind the spine, in the region of the kidneys. Dr. Lippi here again saw a large number of lymphatic vessels terminate in the vena cava, some passing before, some behind the aorta, to reach that vein, and others again entering the splenic and mesenteric veins, which are branches of the vena portæ.

These anatomical facts led Dr. Lippi to think that the theory of venous absorption might be refuted; but the writer before us (M. Defermon) does not think that any person will be led to concur in the opinion of the Italian anatomist. There have been already many communications of the lymphatic vessels with these veins discovered; and these new facts, at most, only lead us to believe that, independent of the imbibition of which the lymphatics are susceptible, in common with all the tissues, these vessels likewise transmit the substances which have been absorbed; but it does not prove any thing against the power of absorption in the veins. Dr. Lippi, in defending the doctrine of Mascagni, shows that he regards the honour of his country more than he does the accuracy of his reasoning.

In the Number of the *Antologia* for November, it is mentioned that Dr. Lippi, in continuing his researches on the lymphatic system, discovered other lymphatic vessels of the mesentery passing to the left side, to empty themselves into the renal veins and their ramifications. The injection which enabled him to ascertain this was passed into the lymphatics of that portion of the mesentery which corresponds to the curvature of the duodenum, where it is tied down to the vertebral column. This new communication of lymphatics and veins seemed to Dr. Lippi to be the course by which substances introduced by the intestines reach the venous system, and also an additional argument against venous absorption.—(***Bulletin des Sciences Medicales***, Dec. 1824.)

PATHOLOGY.

3. M. Hufeland *on Enlargement (Hypertrophy) of the Brain.*—The author of this article, has frequently observed, in making an inspection of those persons who have shown all the symptoms of hydrocephalus, and in whom but an extremely small quantity of serum was found in the ventricles of the brain, that this organ had acquired a much greater volume than the capacity of the cavity of the cranium could properly contain, and in consequence of which it had suffered compression. M. Hufeland inferred from this remark, that the brain, more particularly of infants, was a part of exceedingly active growth, becoming evolved much quicker than other organs. If in this case, therefore, the cranium, from ossification of the sutures taking place too quickly, does not adapt itself to this early development of the brain, the consequence will certainly be compression, which will give rise to all the symptoms characteristic of hydrocephalus. The author goes still further: he thinks that the congestion which results from inflammation, and is the common cause of serous effusion in the brain, is produced by compression of this organ; and, consequently, that acute hydrocephalus is often only a consequence of a similar enlargement *(physconie)* of the cerebral organ.

As to the rest, M. Hufeland only submits these reflections as worthy to excite the attention of the profession; and adds, that, if they shall be confirmed, they will afford an additional proof that it is of the highest importance carefully to attend to the education of infants, both physical and moral, avoiding with care all those causes which would excite too early a development of the brain. Thus, too early exercise of the intellectual functions, the use of spirituous liquors, too highly seasoned food, coffee, &c. should be carefully abstained from. On the contrary, we must assist the development of muscular action, directing frequent ablutions of cold water to the head, and prescribing from time to time slight purgatives: these will prove, according to M. Hufeland, the best preservatives against this disorder, which is now, alas! too frequent.—*(Bulletin des Sciences Medicales*, Decembre.)

4. M. Scoutetten *on Enlargement of the Brain.*—Antoine Peisset, a young lad, five and a half years old, born of healthy and sound parents, exhibited an extremely large head, equalling in size that of a strong healthy adult; the development of which had proceeded so imperceptibly, as not to furnish his parents with any idea as to the time it first began to enlarge. The forehead was elevated, but not prominent: the protuberances on the os occipitis had attained a very large size. For some time, this boy did not complain of any pain; he was not constrained to keep quiet by any other cause than the weight of his head, and, when he wished to walk or run, he suddenly threw himself forwards, and then generally fell. These inconveniences, however, did not seem to increase; and, during the last year, he frequently presented himself at the military hospital of Metz. There was nothing remarkable in his intellect; he understood and remembered very well what was said to him, but he did not recollect any occurrence of his previous life.

When he sat down and remained quiet, he frequently fell asleep, but otherwise he had no remarkable heaviness. All the functions of young Peisset were executed naturally; and, without any cause appearing to give rise to it, suddenly they observed, in the beginning of September, 1823, his appetite fail, at the same time that he was very thirsty, and had a sort of flying pain in the epigastric region. The belly still remained soft; his pulse was hard, full, and frequent; but still they did not perceive any alteration in the functions of the brain, or in the head, although there was certainly a degree of heaviness, from which he was directly roused on speaking to him. His stools were abundant, favoured by the exhibition of castor-oil twice, and by emollient enemas. These means caused the evacuation of several large worms; but in other respects he was not much relieved. During fifteen days, the morbid phenomena did not experience any material change. On the sixteenth, the patient was suddenly, (and for which no cause could be assigned,) seized with an aggravation of his symptoms. The functions of the brain became entirely abolished; the pupil was dilated, although the iris still retained the power of contraction in a high degree; his respiration was embarrassed; his pulse flagged, and became soft; and, about four o'clock in the afternoon, he expired.

*Inspection of the body.*—External appearances: Head extremely large, and very much resembling that of an adult of large stature; the posterior part very greatly developed; the eyes sunk in the orbits; the belly slightly enlarged; the inferior extremities rather small and thin.

*Inspection of the head.*—The skull was from a line and a half to two lines in thickness; the places where the sutures are situated did not appear thinner than the other parts; the meninges of the brain adhered very closely to the skull, and required rather powerful efforts to detach them. Mr. Scoutetten did not remark any alteration in the structure, but the vessels were distended with blood; the membrane was extremely red all over, and showed in many points very distinct exudations of blood; as also many white patches, formed by the thickening of the parts. The plexus choroides was at the same time highly injected. The brain was greatly enlarged, and exhibited throughout its structure much greater density than is generally met with in persons five years of age; its structure was of reddish colour, but did not show any signs of morbid alteration. The enormous development of the brain seems to have taken place chiefly on the upper parts and sides of the hemispheres. He now made a perpendicular incision into the ventricles, of the depth of three inches, leaving only about an inch of the base undivided. The cavity of the ventricles contained only a very small quantity of reddish serum.—(*Archives generales de Medecine,* January.)

5. *Case of diseased Spinal Marrow.*—Madame Marten Charlet, a widow, thirty-six years of age, a native of Paris, of ordinary stature, of a cold, nervous temperament, was ill of what was considered as a nervous complaint, when she was about twenty-seven years old; since which time until she was thirty-four, she had enjoyed good health. At this time, however, she was obliged to work at her needle for subsistence, in consequence of which she generally sat up very late at night. She lived in a low damp situation, and frequently felt the cold (to use her own expression) strike to her kidneys. A short time afterwards she was seized with a severe pain in the right arm, to remove which she made use of a great variety of means; but it still continued, and she could only find relief from opiates. The pain soon after became accompanied with head-ache; the menses had ceased. A month afterwards she came to the hospital. The pains in the arm had increased, and the power of motion had gradually become lost. Convulsions had now attacked the lower extremities, followed by complete paralysis. When she arrived, the patient appeared of a regular and natural figure; her mental faculties perfectly sound. She did not complain of much pain; neither did she suffer much in the left arm, although it was motionless. The motions of the right, although difficult, could, however, be performed. This last had now become the seat of great pain. She had but little appetite, and some thirst. The tongue was slightly red; her respiration easy, but weak; her pulse sometimes frequent and full, but in general regular and small. She had a large and deep slough on the sacrum; the lower limbs were œdematous; the lower part of the chest, and the organs lower down which are subject to the will, had the faculty of feeling and motion entire; at the same time, the patient complained of darting twitches through the belly, which gave the sensation of extreme cold. The urine and fæces passed off involuntarily.

Two blisters were applied to the vertebral column, at the same time that emollient drinks were ordered; she was also ordered a generous diet. The slough on the sacrum was dressed daily, at first with a plaster and lint, afterwards with pledgets spread with styrax. In this way she continued gradually and almost insensibly sinking, and without any remarkable new symptom, until the 2nd of December, when the slough had destroyed all the integuments covering the sacrum, and also part of the haunches; but without having at any time caused the patient much pain. The power of motion in the right arm was insensibly lost. She again complained of severe pain in the arm, of a pricking or pinching nature, and so severe as to make her cry out. The weakness increased very perceptibly, and a feverish state came on; her voice almost failed, her eyes gradually closed; and she died, after having been two months and a half in the hospital, without much agitation or apparent agony.

*Inspection.*—The lower limbs were œdematous, and there was nothing else remarkable in the external parts. In the head, the brain was very sound, and of good consistence. When the vertebral canal was first looked at, there did not appear to be any mischief; but, as soon as the dura mater was opened, we saw upon the tunica arachnoidea, for full three-quarters of its lower part, a great number of small tubercles *(plaques)*, of a whitish opaque colour, the diameter of which varied from three to four lines; they were more than a quarter of a line in thickness; they were chiefly in the posterior part of the canal, only a few, very small, ones remaining in front; the surface opposed to the spinal marrow was rough and irregular, the other was smooth and polished. They did not adhere either to the spinal marrow or pia mater, but seemed suspended in the tunica arachnoidea, which was transparent and quite sound round these tubercles. The medulla spinalis was also sound, but rather more firm than natural; the rest of the membranes were not at all thickened. The posterior origins of the nerves were distinct and natural: the whole of them were not seen,—only those on the posterior part. The marrow also, cut into in several places, and seen posteriorly, did not show any thing remarkable; but, on lifting it from its canal, we discovered, high up in the dorsal region, a foreign production, which covered the whole of the anterior part of the medullary cord, from the sixth cervical pair of nerves to the third dorsal.

The colour of this substance was a reddish yellow, and it was situated between the spinal marrow and the arachnoid tunic, in such a way as strongly to compress the former, with which it seemed intimately connected. It was impossible to distinguish the proper membrane of the spinal marrow. This substance was flatter and thicker on the left than on the right side: in the first, viz. the left, being rather more than three lines in thickness; whereas, in the second, it did not exceed one half. This last portion on the right side could be turned over towards the left, as far as a sort of line, from which it seemed more directly to derive its origin, and where the roots of the corresponding nerves could not be in the least distinguished. The posterior roots of this side were visible, but those of the right were certainly altered. The anterior fibres were compressed to such a degree, that only a few filaments could be discovered. The medulla spinalis was much flattened by this substance, but more particularly on the left side; the nature of it seemed to be very like that of the cerebrum; its surface was soft and unequal, and having a slight appearance of folds, of a reddish colour; its form irregular and flattish; and indeed it had very much the external appearance of the medulla spinalis, covered with its proper membrane. Its texture was neither fibrous nor cartilaginous; neither was there indication of tubercles or schirrus. Its substance seemed to be of a fatty nature, in which we could see vessels and cellular membrane; or, in short, it showed the same consistence and characters as that texture, to which it was connected, and of which it was evidently a continuation.

Both above and below this substance, the medulla spinalis was extremely healthy, and, although cut into in several places, it did not show the least morbid change. The slough on the sacrum was not at all connected with the interior of the vertebral canal.

M. Jolly has taken two separate views of this case, and the morbid parts are preserved in the museum of the Faculté de Medecine.—(*Archives generales,* January.)

6. *Cicatrization of the Heart.*—M. Bougon has presented to the Royal Academy of Medicine, the heart of a person who died in his hospital. This person showed very distinct marks of an old wound, which had penetrated the chest, and in which the lung, pericardium, and heart, were wounded. All these organs were cicatrised. This person died of disease not at all connected with the wound above mentioned.—(*Ibid.*)

### THERAPEUTICS.

7. *Accidents arising from Acupuncturation.*—M. AUMONT has related some remarks on the case of an officer, on whom he had practised acupuncturation of the abdomen, for extremely severe pains situated there, and which he could not relieve by any means he had used. The first needle was introduced two fingers' breadth from the umbilicus, and caused great pain; the second was then passed on a level with the first, along the inner edge of the muscle on the right side. This second puncture was scarcely made, when the patient fainted; and, on recovering himself, he was afflicted with very excruciating pain and heat in that part of the abdomen, which were quickly followed by general fever. The patient came into the Val de Grace, where this state lasted for some days, and then yielded to an antiphlogistic treatment; but the pains for which he had submitted to acupuncturation were not the least changed, nor did this method at all relieve him.

M. BECLARD mentions, on this subject, that fainting is a common accident from acupuncturation, and which he had very recently observed in the case of an individual, into whose deltoid he had passed five needles, on account of an extremely severe rheumatic pain situated upon his shoulder, and which has since disappeared. He considers the operation as by no means free from risk, and quotes the case of a person into whose leg he had passed a needle, to relieve a severe pain there. No sooner was the needle introduced, (doing which gave great pain,) than the patient was seized with syncope, lasting for some time, and which did not yield without giving rise to a high delirium. This nervous excitement gradually abated, but the patient remained the whole of the day in a state of torpor or dullness, which at length disappeared. An abscess was afterwards slowly formed at the seat of the puncture.—*(Ibid.)*

---

### MIDWIFERY.

8. *Case of a Child's crying while the Head was yet in utero.*—M. MARC lately read, to the Royal Academy of Medicine, the case of a woman, twenty-seven years of age, who had arrived at her full period of gestation, but in whom the pelvis was deformed. At the time the accoucheur was called to assist her delivery, the waters had been discharged twelve hours. He ascertained, by examination, that the head was in its natural position; the mouth of the uterus was dilated to the extent of two inches. The extreme narrowness of the passage obliged him to turn the child, when the trunk and limbs were expelled from the womb, but the head still continued fixed above the narrow pelvis, and in the cavity of the uterus. As soon, however, as he endeavoured to draw down the head, the child cried so powerfully as to be heard by the assistants, and the head still continued fixed; neither could it be perceived at the external orifice. After a short period, he renewed his endeavours to remove it a second time; the child again cried, but more faintly; also a third time, but the cry was still more feeble; and lastly, when the child was brought away, it showed but very slight signs of life, and soon expired.—*(Ibid.)*

### METEOROLOGICAL JOURNAL,

*From January 20 to February 19, 1825.*

By Messrs. WILLIAM HARRIS and Co. 50, Holborn, London.

| | MOON. | Rain gauge | THERM. | | | BAROM. | | DE LUC'S HYG. | | WIND. | | ATMOSPHERIC VARIATIONS. | | |
|---|---|---|---|---|---|---|---|---|---|---|---|---|---|---|
| | | | 9 A.M. | MAX. | MIN. | 9 A.M. | 10P.M. | 9 A.M. | 10P.M. | 9 A.M. | 10P.M. | 9 A.M. | 2 P.M. | 10P.M. |
| Jan. | | | | | | | | | | | | | | |
| 20 | | | 40 | 42 | 39 | 29.48 | 29.52 | 89 | 85 | NW | NW | Cloud. | Cloud. | Cloud. |
| 21 | | | 40 | 41 | 35 | 29.63 | 29.70 | 89 | 90 | NE | N | Fine | Rain | Fine |
| 22 | | | 37 | 39 | 35 | 29.80 | 29.96 | 92 | 92 | NE | NE | Cloud. | Cloud. | |
| 23 | | .08 | 37 | 40 | 35 | 30.08 | 30.03 | 90 | 90 | NE | N | Fine | | Cloud. |
| 24 | | | 37 | 40 | 37 | 29.94 | 29.78 | 92 | 90 | NE | SSW | Cloud. | | |
| 25 | | | 38 | 42 | 33 | 29.53 | 29.79 | 92 | 90 | NNW | WNW | | | |
| 26 | | | 35 | 37 | 38 | 29.92 | 29.87 | 85 | 95 | SW | SW | | | |
| 27 | ☽ | | 42 | 43 | 36 | 29.92 | 29.94 | 95 | 75 | SW | SW | Fog | | |
| 28 | | | 38 | 42 | 33 | 30.40 | 30.56 | 70 | 71 | NE | NNW | Fine | Fine | Fine |
| 29 | | | 34 | 37 | 35 | 30.58 | 30.57 | 75 | 71 | W | WSW | Fog | Fog | Foggy |
| 30 | | .15 | 41 | 45 | 45 | 30.35 | 30.30 | 69 | 74 | WSW | SW | | Cloud. | Cloud. |
| 31 | | | 46 | 48 | 40 | 30.35 | 30.30 | 75 | 71 | SW | SW | | Fine | |
| Feb. | | | | | | | | | | | | | | |
| 1 | | | 47 | 49 | 34 | 30.00 | 30.10 | 72 | 70 | SW | NW | Fine | Cloud. | Fine |
| 2 | | .90 | 35 | 39 | 48 | 30.26 | 29.83 | 70 | 73 | WSW | SW | | Fine | |
| 3 | ○ | | 51 | 48 | 30 | 29.40 | 29.41 | 74 | 68 | W | W | Foggy | | |
| 4 | | | 32 | 33 | 28 | 29.51 | 29.61 | 74 | 70 | WNW | W | Sn.& F | Sn.& F | Cloud. |
| 5 | | .12 | 31 | 35 | 33 | 29.54 | 29.61 | 69 | 69 | NW | NW | Fine | Fine | Fine |
| 6 | | | 35 | 39 | 32 | 29.90 | 30.12 | 65 | 72 | N | NW | | | Cloud. |
| 7 | | | 37 | 38 | 39 | 30.05 | 29.84 | 73 | 77 | SW | SSW | Foggy | | Rain |
| 8 | | .18 | 40 | 43 | 35 | 29.84 | 29.96 | 76 | 72 | SW | NW | Cloud. | | Fine |
| 9 | | | 37 | 44 | 40 | 30.22 | 30.29 | 76 | 75 | W | W | | | Cloud. |
| 10 | ☾ | | 41 | 47 | 35 | 30.32 | 30.37 | 76 | 76 | W | W | Fine | | Fine |
| 11 | | | 40 | 46 | 35 | 30.40 | 30.41 | 77 | 75 | W | SW | Foggy | | Cloud. |
| 12 | | | 40 | 41 | 31 | 30.41 | 30.41 | 76 | 76 | W | W | | | Fine |
| 13 | | | 36 | 40 | 37 | 30.42 | 30.17 | 76 | 77 | WSW | SSE | | | Foggy |
| 14 | | .10 | 39 | 42 | 39 | 29.43 | 29.92 | 76 | 76 | SSE | S | | Cloud. | Cloud. |
| 15 | | | 41 | 47 | 45 | 29.87 | 29.90 | 76 | 76 | SW | SW | | | |
| 16 | | | 49 | 49 | 44 | 29.88 | 29.83 | 77 | 79 | SW | SW | Fine | Fine | |
| 17 | ● | | 45 | 47 | 43 | 29.83 | 30.02 | 77 | 76 | E SE | E | Sleet | Sleet | |
| 18 | | | 44 | 46 | 44 | 30.06 | 30.10 | 78 | 80 | E | SE | Cloud. | | |
| 19 | | .06 | 45 | 47 | 44 | 30.12 | 30.12 | 76 | 78 | SSE | SW | | Fine | |

The quantity of rain fallen in the month of January, was 77.100ths of an inch.

